# Essential Role of Icosahedral Symmetry in the 3D Shape of AuAg Plasmonic Nanostars With High Aspect‐Ratio Legs

**DOI:** 10.1002/smll.73216

**Published:** 2026-03-29

**Authors:** Leonardo M. Corrêa, Simon M. Fairclough, Kaleigh M. R. Scher, Supriya Atta, Diego P. Dos Santos, Caterina Ducati, Laura Fabris, Daniel Ugarte

**Affiliations:** ^1^ Universidade Estadual de Campinas Instituto de Física Gleb Wataghin Campinas São Paulo Brazil; ^2^ Department of Materials and Metallurgy University of Cambridge Cambridge UK; ^3^ Department of Materials Science and Engineering Rutgers University New Jersey USA; ^4^ Department of Biomedical Engineering Duke University Durham North Carolina USA; ^5^ Instituto De Química Universidade Estadual de Campinas Campinas Brazil; ^6^ Department of Applied Science and Technology Politecnico di Torino Torino Italy

**Keywords:** 4D‐STEM, crystal orientation mapping, morphology, nanostars, plasmonic nanoparticles, precession electron diffraction

## Abstract

Plasmonic nanoparticles play an essential role in improving the sensitivity of different optical methods, and multibranched nanostars are predicted to show the highest enhancement factor for several applications in the near infrared region. The structural complexity of these particles represents a serious challenge with respect to gathering precise knowledge of atomic arrangement and 3D star morphology, making it difficult to accurately predict their optical response and to devise new synthetic methods addressing various applications. Here, we have used 4D‐STEM diffraction mapping and a thorough crystallographic analysis to determine leg configuration in space, so that a better understanding of plasmonic properties and growth mechanism can be obtained. Our results show that although electron microscopy images may show a certain shape diversity with stars displaying 4‐ 5‐ or 6 branches, the orientation of these legs in space follows the positions associated with the apexes of an icosahedral core. The direct application of the measured star structures has been used as quantitative structural input in optical simulations, which show excellent agreement with experimental measurements. This evidence confirms the crucial role of icosahedral symmetry for explaining the growth mechanism and plasmonic response of gold nanostars with high‐aspect‐ratio legs.

## Graphical abstract

Electron nanodiffraction mapping was used to determine that the 3D leg configuration of branched plasmonic AuAg nanostars is associated with the icosahedral symmetry of the star core, providing a deeper understanding of their growth mechanism and thus paving the way to the development of optimized synthesis protocols targeting optical enhancement effects for their application in the near infrared region.

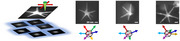

## 1 Introduction

The development of nanotechnology has led to new materials and original devices by improving synthesis, manipulation, processing, and access to precise structural, physical, and chemical characterization methods with high spatial resolution [1, 2]. The exploration of localized surface plasmon resonances (LSPR) represents a key example of nanomaterials optimization [3]. These collective electron excitations are strongly dependent on nanoparticle (NP) size, morphology, and spatial arrangement, so the optical response can be tailored to drastically increase sensitivity in optical spectroscopy measurements. Surface‐enhanced Raman scattering (SERS)‐based methods represent a typical example of LSPR manipulation and exploration of nanoparticle plasmonic response [4]. The progress in wet chemical synthesis methods has brought NP synthesis to an astonishing level of control toward the bottom‐up construction of nanosystems [1]. At present, it is possible to produce multidomain and multielement particles to achieve pre‐determined chemical or optical properties with high reproducibility. These particles may be core‐shell structures of different geometrical shapes (spheres, cubes, bipyramids) or even multibranched complex morphologies [5, 6, 7, 8, 9, 10, 11], of which branched nanostars (NS) with high aspect ratio legs synthesized by a seeded growth procedure represent one of the most refined and interesting wet chemical synthesis achievements [12, 13, 14, 15]. However, we must emphasize that the accurate measurement of atomic arrangement and 3D morphology of nanoparticles showing a complicated chemical and geometrical composition is quite challenging and represents yet an unresolved issue. It is essential to gather detailed knowledge on the crystallographic and chemical structure of these complex NPs to develop a robust understanding of their growth mechanism and of the different steps of their synthesis protocols.

Transmission Electron Microscopy (TEM) and the corresponding scanning mode (STEM) are the most popular experimental tools to characterize nanomaterials with high spatial resolution [16, 17, 18]. Any transmission electron microscope (TEM or STEM), however, provides images only containing the 2D projections of nanoparticles; to gather information of 3D shape, discrete electron tomography (ET) must be applied [19, 20, 21, 22], which requires the acquisition of a series of several tens of images at different sample tilt angles in relation to the incident electron beam (or the projection direction). ET based on High Angle Annular Dark Field (HAADF) STEM has been exploited for several years to obtain information on particle faceting or atomic details of atomic arrangement inside NPs [19]. It is important to mention that STEM ET remains quite demanding, considering electron irradiation doses of 10^5^–10^6^ e^−^Å^−2^ (electrons per square Angstrom) for the acquisition of 50–70 images and ∼30–60 min total irradiation time. Therefore, this method must be applied with caution for beam‐sensitive samples to avoid sample modification by radiation damage effects.

The rapid development of instrumentation in the field of electron microscopy (optics, detectors, automation, etc.) has generated many different scanning TEM approaches to map structural, chemical, and physical properties of materials [2]. Among these methods, electron nanodiffraction mapping (usually called 4D‐STEM) is constantly increasing in popularity [23]. By scanning a few‐nm‐wide electron beam over a sample and recording the diffraction pattern for each pixel position, we can map the atomic arrangement with nanometer resolution. In comparison with atomic resolution images, 4D‐STEM studies display several advantages; the main one is a substantial reduction in electron dose, which allows the study of atomic arrangement in beam‐sensitive materials [24, 25, 26, 27]. In addition, diffraction signals generated by nanocrystalline regions show high intensity peaks on a slowly varying background, so the signal‐to‐noise ratio is usually intrinsically very high and requires short dwell time per pixel.

Using 4D‐STEM, the atomic arrangement of nanomaterials can be obtained without the need of an electron beam with angstrom or sub‐angstrom width, so that the total electron dose is lowered by several orders of magnitude to approximately tens or hundreds of e^−^Å^−2^. [24, 25, 26, 27, 28] In addition, diffraction maps are acquired using a few‐nanometer‐wide beam (1–10 nm in diameter), thus not requiring complex and expensive aberration‐corrected transmission electron microscopes (AC TEMs [2]). From a different viewpoint, the use of few‐nm‐wide beams allows the analysis of larger fields of view over the sample, providing more statistical validity to the structural study. In contrast, atomic resolution images must be usually considered local measurements due to the rather small sample area analyzed (a few tens of nm). Finally, a 4D‐STEM data set usually includes a huge number of patterns; for example, a 100 by 100 pixels scan will generate 10 000 diffraction patterns, and must be analyzed with big data methodologies, such as machine learning and artificial intelligence tools to extract relevant information [29].

**FIGURE 1:**
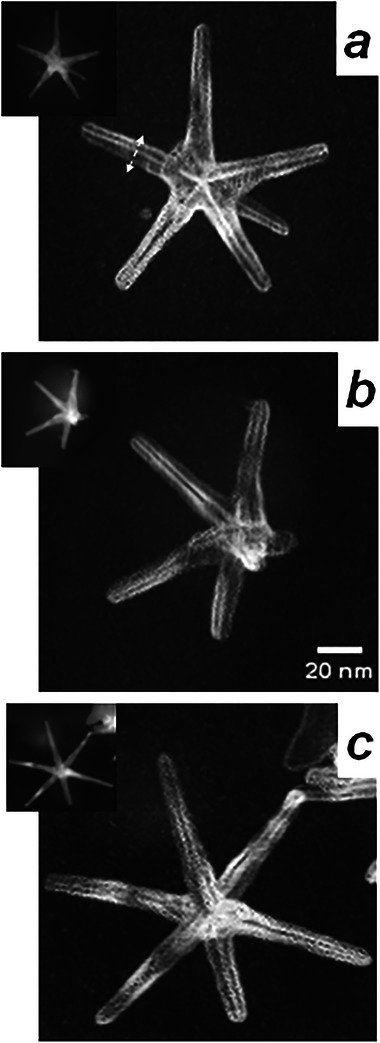
Virtual anticorrelation image revealing abrupt crystallographic changes between pixels (1 nm pixel step) for NS with different legs configuration (a) NS‐5, star showing legs in five‐fold symmetry; b) NS‐4, anisotropic star where the image allows the straightforward identification of 4 high aspect‐ratio legs; c) NS‐6, star displaying 6 legs, which has been determined to present a rather planar spatial leg distribution and an icosahedral core by Corrêa et al. [28] Note that bright lines (low similarity between neighboring diffraction patterns) are observed along the legs, revealing their multiple‐twinned decahedral structure; leg axis follow *<010>_BCO_* crystallographic direction (*BCO*, Body Centered orthorhombic), what allows a direct identification of legs orientation in space after an ACOM analysis of crystal region inside legs. Insets show VADF STEM images of the NSs, which are very useful to visualize star morphology but provide little useful information on leg internal structure.

A 4D‐STEM dataset can be exploited to generate a series of virtual images whose contrast is associated with different structural and crystallographic details (spatial information or maps that are calculated by post‐processing) [30]. For example, conventional Annular Dark Field (ADF) STEM images are generated by analyzing the diffraction pattern (DP) for each pixel and integrating the recorded signal between a selected scattering angular range (so‐called virtual ADF (VADF), see Figure 1). When a particular diffraction beam is selected to build the image intensity, we may map the sample regions being the source of the diffracted spot (i.e. Virtual Dark Field, VDF), which is very useful to identify the spatial distribution of similarly oriented crystals.

Crystallographic information carried by an electron diffraction (ED) pattern may be very detailed [16, 17, 18]; however, it may not be straightforward to extract and analyze. ED is in many aspects quite different from x‐ray diffraction: the strong interaction cross‐section of electrons with matter leads to a so‐called dynamical regime, where data interpretation may require complex and time‐consuming numerical calculations [16, 17, 18]. During the last decade, the development of Precession Electron Diffraction (PED) has transformed ED into a different diffraction tool described as operating in a “quasi‐kinematical” regime, where diffracted beams can be correctly modelled using the much simpler kinematical approach routinely applied in x‐ray diffraction [31, 32]. PED mapping has become very popular during the last two decades due to its application in association with a nanometer‐wide beam and Automatic Crystal Orientation Mapping (ACOM) software [33, 34, 35], which enables efficient and precise measurements of crystal texture or strain in metal and semiconductor systems. Recently, the exploration of PED‐based ED has revealed many new opportunities to gather atomic structure details and 3D morphology in individual nanoparticles and nanowires [28, 36, 37, 38, 39, 40].

Multibranched AuAg nanostars with high aspect ratio legs have been predicted to have the highest scattered electric field enhancements, which may in turn be reflected in SERS enhancement factors [41]. In addition, the high aspect ratio legs, which resemble nanowires, generate plasmons in different infrared windows [15]. Because of their optical activity in the near infrared, they show high potential for applications in biodetection [42]. Because nanostar LSPR modes should arise from the coupling of charge oscillations in the different NS legs, a thorough understanding of the physical origin of the LSPR modes requires the precise determination of the leg distribution in space. Herein, we have used PED‐based 4D‐STEM diffraction mapping to determine the atomic structure and 3D shape (leg orientation in 3D space) of NSs through a crystallographic analysis of the nanostar core and legs. This includes analyzing the wider range of information available from a 4D‐STEM diffraction data set: i) leg positions in virtual STEM images (2D, azimuthal angular data, VADF and VDF); ii) symmetry of core electron diffraction patterns; and iii) leg elevation angles (3D configuration) through crystal orientation mapping (ACOM). Also, as the study is based on crystallography analysis, leg orientation is derived as a set of vectorial entities, allowing their direct quantitative use as a structural model for optical simulations, which show excellent agreement with experimental measurements. The application of 4D‐STEM greatly decreases the risk of electron radiation damage, minimizing possible modification of NSs, especially when compared to STEM tomography observations, which involve high‐dose data acquisition.

## 2 Results and Discussion

Low magnification images of the NS sample reveal that most stars show 4, 5 or 6 high aspect‐ratio legs with diameters in the 7–10 nm range [14, 15, 28]. Due to the inherent bidimensional projection character of TEM images, the azimuthal position of legs can be measured, but the arrangement of legs in 3D space cannot be directly inferred from these images. Many stars with 5 and 6 legs display inter‐leg azimuthal angular distance of 60–70 degrees, which may suggest 6‐fold or five‐fold symmetry of leg configuration (Figure S1). Figure 1 reveals the VADF STEM images measured for three different NS morphologies (hereafter named as NS‐5, NS‐4, and NS‐6, respectively), which are frequently observed in low‐resolution TEM images.

The leg configuration for NS‐6 (star showing 6x major legs) has been thoroughly investigated using 4D‐STEM diffraction mapping by Corrêa et al. [28]. Their study analyzed the nanostar under different orientations relative to the electron beam (0° and 10° tilt), providing robust insights into both the core structure and the 3D arrangement of the legs. Based on VDF imaging, the core was established to be an icosahedral (ICO) particle, while the branches display decahedral (DEC) wire structures. Atomic resolution TEM images [14] had previously suggested that the legs were decahedral multiple‐twinned rods, an interpretation further validated through detailed diffraction mapping across the nanostar branches (see ref. [28]). Crystal orientation measurements revealed that NS‐6 displays a rather planar spatial distribution of the legs, with their positions and orientations corresponding to the directions of the five‐fold axes at the apexes of the ICO core. This 3D spatial information was directly derived from PED mapping utilizing established ACOM methodologies [33, 34, 35].

The results mentioned above explain why the atomic arrangement in the nanostar showing high aspect‐ratio legs has been so challenging to understand from TEM images or ensemble powder diffraction methods. The structure is based on the assembly of two different kinds of multiple‐twinned structures: icosahedra and decahedra. The icosahedral core shows a slight distortion from the equilibrium *FCC* (Face Centered Cubic) phase to Rhombohedral (*RHO*) symmetry, while the decahedral legs exhibit a Body Centered Orthorhombic (*BCO*) structure. [28, 43] Leg axes align along the *[010]_BCO_* direction, which facilitates their identification using ACOM crystal orientation measurements, as detailed by Corrêa et al. [28].

The analysis of polycrystalline nanoparticles can be greatly enhanced using anticorrelation maps (ACM, see Supporting Information), where image intensity is obtained by analyzing the similarity of a pixel diffraction pattern with patterns from neighboring pixels [44]. These images highlight grain boundaries and twins with higher intensity, since they correspond to sudden changes in crystal phase or orientation. All three nanostructures present ACM images that confirm the presence of grain boundaries (twins) along each leg, suggesting that all high aspect‐ratio legs are DEC multiple twinned rods.

**FIGURE 2:**
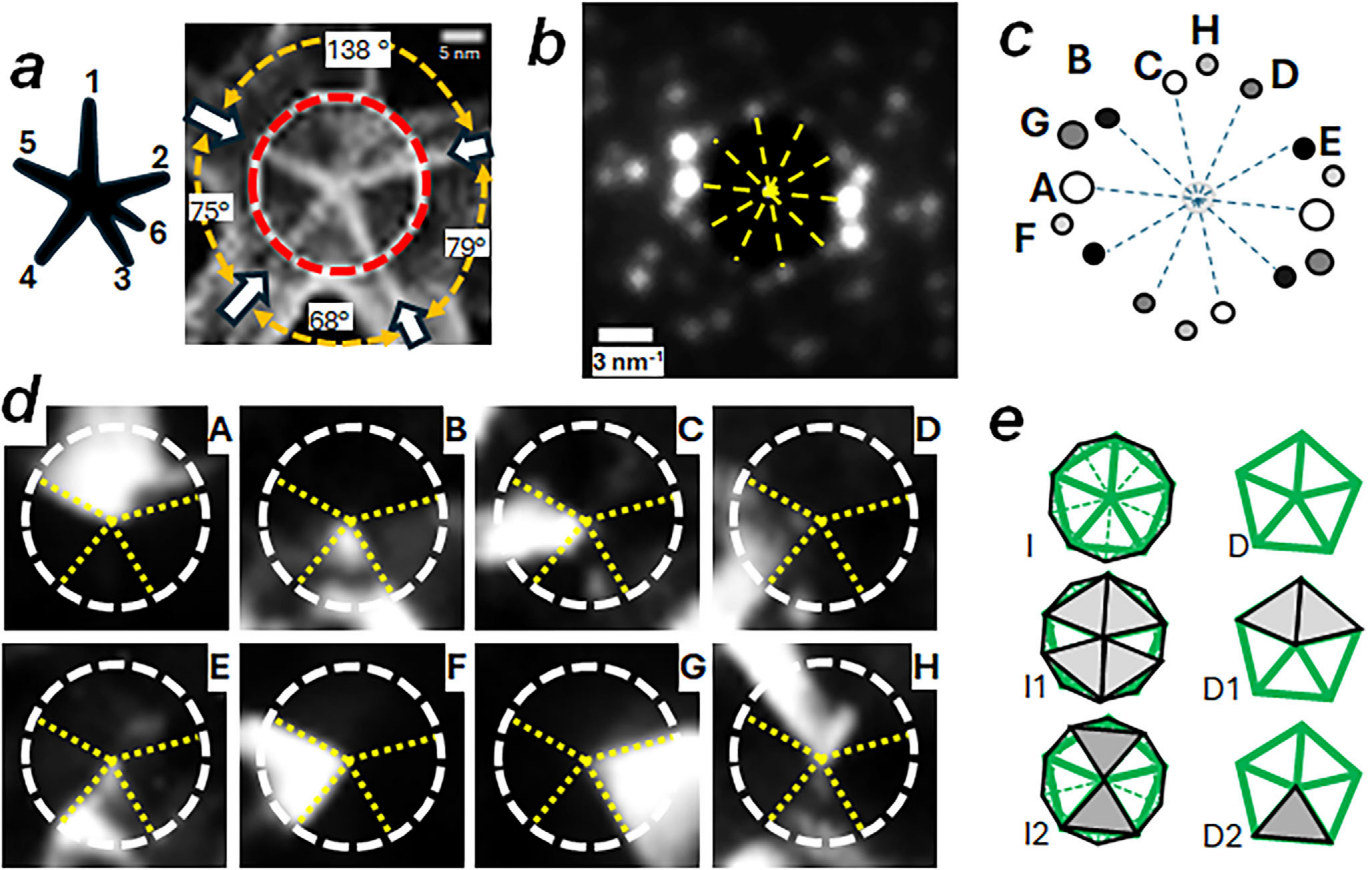
(a) Closer view to the anticorrelation image of NS‐5S5 showing the high intensity lines (indicating twin positions) join at the NS core with approximately five‐fold angular distribution (measured angles between bright lines are shown; red circle indicates a 20 nm in diameter). b) Mean diffraction pattern from the NS central region (pixels inside red circle in (a)). Note the 10‐fold rotation distribution of spots as indicated by dashed lines (schematic drawing of the core ED shown in (c)). d) VDFs derived from diffraction spots marked A‐H (white dashed circle diameter is 20 nm, and spotted lines indicate leg twin direction as revealed by ACM). e) Expected VDF contrast patterns generated from the decahedral and icosahedral geometrically perfect nanoparticles when observed along the five‐fold axis; grey areas represent the expected high intensity regions revealing crystal domains originating the diffraction spots. Most of the VDFs show a single or two crystal domains well delimited by twin positions as expected by the decahedral structure. VDF from spot H can only be explained by the icosahedral model (scheme indicated as I2 in (e), two triangular regions connected by an apex at the core centre). See text for explanations and schematic drawing of tetrahedra ordering in icosahedral particles in Figure S4a.

The STEM images presented in Figure 1a  reveal that NS‐5 exhibits an azimuthal leg distribution closely resembling five‐fold symmetry. The ACM image of NS‐5 high‐intensity lines, which indicate twin defects along the legs that converge at the center of the star core (Figures 1a and 2a). This information is highly significant, as it highlights a clear interdependence between the spatial orientation of the legs and the structure of the star core.

To obtain information on the core structure, we have analyzed the mean diffraction pattern obtained by adding the DP at pixels located in the central region of the particle (Figure 2b,c); this DP shows a family of spots distributed with 10‐fold symmetry. Observation of five‐fold rotation symmetry represents an unusual and singular event in crystallography. In nanoparticle research, two categories of multiply twinned particles (MTPs), decahedra and icosahedra, show this symmetry [45]. Both MTPs have been associated with the nanostar core structure [11, 15, 28]. DEC and ICO NPs have a unique crystal domain organization that will provide very distinct VDF when selecting equivalent diffraction spots; we show the expected VDF contrast patterns for both particles in Figure 2e. VDFs have been calculated using diffraction spots marked A‐H (Figure 2c); most of these images reveal crystal domains delimited by the grain boundaries appearing as high intensity lines in the ACM image (Figures 1a and 2a). Key information on core symmetry can be obtained from the VDF generated by spot H, which shows two triangular regions with an apex connected at the particle's center (sand clock configuration). This contrast pattern can only be generated by an ICO core (see Supplementary Information, Figure S4a); an identical procedure has been used by Corrêa et al. [28] to determine the icosahedral symmetry of the NS‐6 core (Figure 1c). The Supplementary Information contains an in‐depth, step‐by‐step explanation of the logical process used for symmetry analysis of images and diffraction patterns obtained from the 4D‐STEM map.

The five‐fold symmetry aspect of the NS‐5 core diffraction pattern (Figure 2b,c) points out to an ICO core oriented along a five‐fold axis. When an icosahedron is observed along the five‐fold direction, some constituent tetrahedra can also be grouped to form two decahedral particles, stacked along the five‐fold direction and rotated by (2𝜋⁄10) in relation to each other (see Figure S4). Any of these decahedra may provide 5 apexes distributed azimuthally at 72‐degree angles, which may serve as an attachment point for the major legs of NS‐5.

Another characteristic of NS with major legs in a five‐fold configuration is the occurrence of a shorter sixth leg at the azimuthal angle between two major legs [14, 28]. A model considering a DEC core does not easily account for or predict a reasonable, specific attachment site of a sixth leg. In contrast, an icosahedral core model offers a simple, straightforward explanation. If leg attachment points are situated on the ICO apexes, it becomes possible to position five primary legs on the apexes of one decahedron, while the sixth leg attaches to an apex from the second stacked decahedron, which is a 36‐degree rotated decahedron along the observation five‐fold axis direction. (see Figure S4).

It is important to mention that we can use a second very stringent test to discriminate if the NS core shows DEC or ICO symmetry: leg elevation angles. According to the DEC‐based NS model, the 5x major legs are expected to emerge from twin planes, growing perpendicular to the five‐fold axis, i.e., with zero elevation angle relative to the sample plane (REF. [14, 15]). For an ICO core and a leg distribution following the icosahedral apex direction (as schematized in Figure S4c) all legs should show similar elevation angles, around ∼**27°**.

**FIGURE 3:**
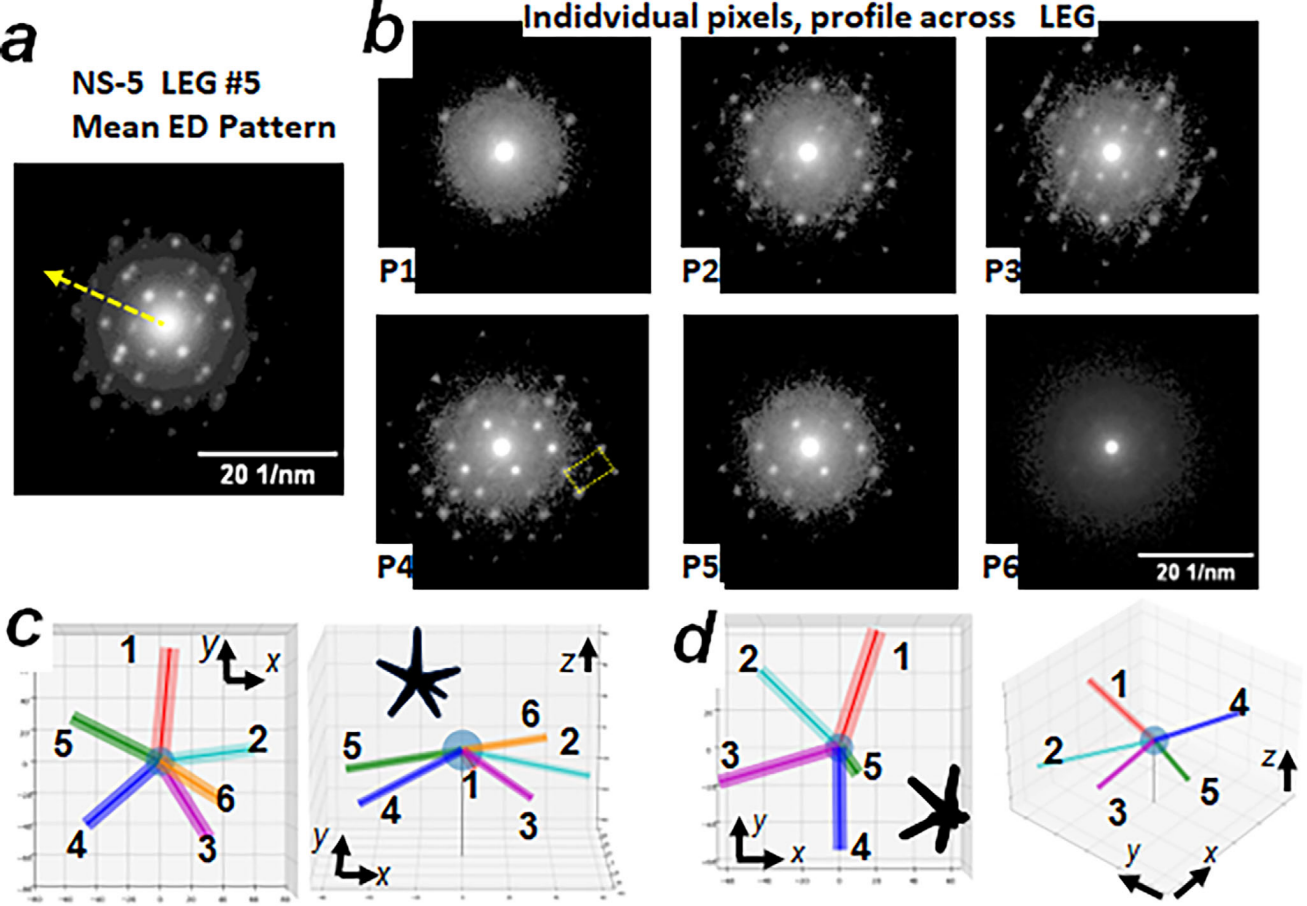
a) Mean diffraction pattern calculated from leg #5 of NS‐5 (base‐tip direction of the leg is indicated by an arrow). b) diffraction patterns extracted from individual pixels along a perpendicular line to leg #5 of NS‐5 (this leg in ∼10 in diameter, pixels are located close to the upper region of the leg). Note the high quality of the recorded ED, showing well‐defined rectangular ED spot distribution in ED from pixels #4 and #5 (P4, P5). By applying template matching ACOM, it becomes easier to assess the orientation of the leg in space.c) and d) Orientation of the legs deduced from ACOM for NS‐5 and NS‐4, respectively. At right, we show the distribution as observed along the electron beam direction, indicating the legs' azimuthal distribution. At left, we show a side view to better observe the elevation angle of the legs (z axis points upward in the plot). Grid at the background represents 20 nm x 20 nm squares.

As our 4D‐STEM diffraction mapping is based on precession electron diffraction, the application of the well‐established ACOM methods [33, 34, 35] can be used to analyze leg orientation in space (decahedral legs axis should follow a *[010]_BCO_* direction [28]). Each decahedral leg is composed of 5 different crystallites joined at the wire axis. The mean DP of a leg (a nanowire ∼7‐10 nm in diameter) is difficult to interpret because it is formed by the superposition of 5 crystals with different orientations. Nevertheless, the interpretation can be significantly simplified by observing diffraction patterns for individual pixels across each leg (Figure 3a,b; Figures S2 and S3). Due to the attained high spatial resolution (2‐nm‐wide electron beam), it is possible to obtain individual pixels from the diffraction map containing information from an isolated crystal inside the legs. The determination of its orientation provides sufficient information of the leg orientation as the other crystallites are bound to the five‐fold organization by its common *[010]_BCO_* axis. The ED from these few pixels yields a clean and well‐defined geometric pattern of diffraction spots (see DP indicated P4 in Figure 3b; Figures S2 and S3), such that crystal orientation information can be efficiently estimated with high angular precision (2‐3 degrees). ACOM results yield the azimuthal and elevation angles from the *xy*‐plane perpendicular to the e‐beam direction (considered here to travel along (*‐z*) direction, Figure 3c).

At this point, it is important to mention that the determination of leg orientation in space suffers an intrinsic degree of freedom due to the bidimensional nature of electron diffraction. This uncertainty is called “180 duality” in ACOM methodology [33]: the absolute value of the elevation angle can be estimated, but it is not possible to determine if the elevation angle is positive (pointing upward (+*z)*, toward the electron beam source) or negative (downward (*‐z*)). To carry out our analysis, we consistently started by assigning leg elevation angles with the same sign (all positive or all negative). Afterward, the elevation angle of certain legs may be inverted (from +z to ‐z or vice versa) to prevent the presence of very small inter‐leg angles (around 20–30 degrees). TEM images almost never display primary legs with such narrow inter‐leg angles.

The elevation angles of all six legs of NS‐5 are similar, ranging from 17 to 22 degrees (see Table S1). Since none of the legs is perpendicular to the five‐fold axis, it eliminates the DEC core model as a possibility for NS‐5. Based on the configuration shown in Figure S4c and the leg orientation convention previously described, we have designated the major legs (1‐5) as pointing downward, while the sixth leg points upward (see experimental ACOM deduction in Figure 3c). |The deduced interleg angular distances in 3D space have been included in Table 1.

**TABLE 1:** Comparison between inter‐leg angular distances in space as deduced from experimental ACOM and geometrically perfect icosahedral symmetry. The three charts show measurements in the lower left sector, while the upper right sector displays ideal values deduced from ideal icosahedral geometry. Note the excellent agreement of the angular values for the three reported nanoparticles (NS‐6, NS‐5, and NS‐4).

**NS‐6 (6 Legs, data from Ref. Correa et al. [** 28 **])**						
**Leg #**	**1**	**2**	**3**	**4**	**5**	**6**
1	**0**	***64.3***	***116.6***	***180.0***	***116.6***	***64.3***
2	57	**0**	***64.3***	***116.6***	***180.0***	***116.6***
3	121	77	**0**	***64.3***	***116.6***	***180.0***
4	157	129	52.4	**0**	***64.3***	***116.6***
**5**	115	171	111	60	**0**	***64.3***
6	56	110	160	121	61	**0**
**NS‐5(5 Major Legs, five‐fold rotation profile)**						
**Leg #**	**1**	**2**	**3**	**4**	**5**	**6**
1	**0**	***64.3***	***116.6***	***116.6***	***64.3***	***116.6***
2	73	**0**	***64.3***	***116.6***	***116.6***	***64.3***
3	127	62	**0**	***64.3***	***116.6***	***64.3***
4	119	130	76	**0**	***64.3***	***116.6***
5	64	133	135	64	**0**	***180.0***
6	121	51	42	111	174	**0**
**NS‐4 (4 major Legs, anisotropic profile)**						
**Leg #**	**1**	**2**	**3**	**4**	**5**
1	**0**	***64.3***	***116.6***	***116.6***	***116.6***
2	65	**0**	***64.3***	***116.6***	***116.6***
3	123	68	**0**	***64.3***	***116.6***
4	132	132	67	**0**	***116.6***
5	108	104	114	110	**0**

**FIGURE 4:**
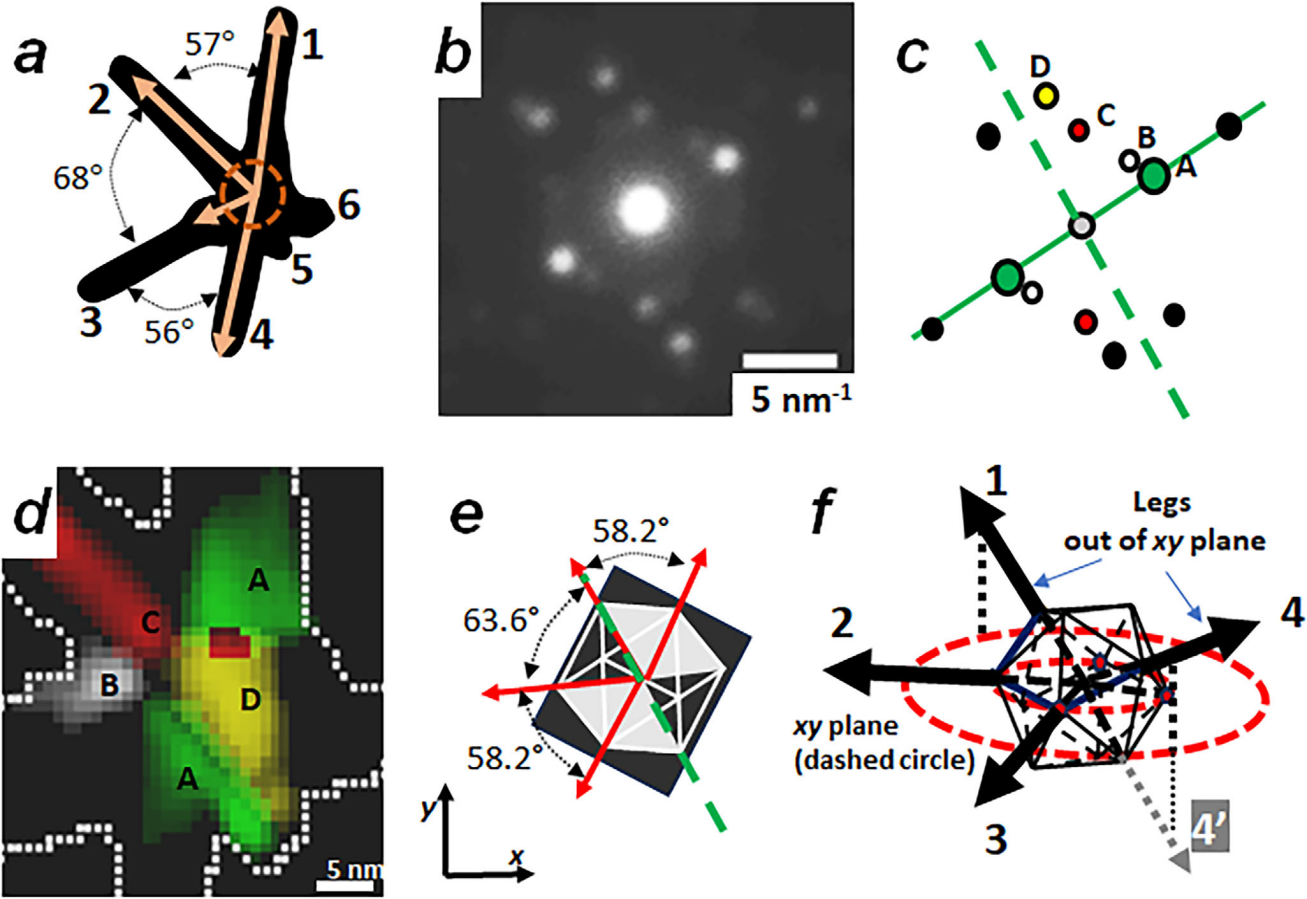
(a) Schematic drawing of highly asymmetric star NS‐4, where 4 high aspect‐ratio legs are clearly recognizable. b) ED pattern from the NS core generated (pixels inside circle included in (a) (20 nm in diameter). Note the rough 2‐fold symmetry of the pattern, whose schematic draw is shown in (c). d) superposed VDF images indicating the spatial distribution of pixels originating diffraction spots marked A‐D (white dotted line indicate NS border); note the clear polycrystalline nature of the core. The stronger diffraction spot (marked A in (c)) corresponds to the *(011)_RHO_* planes (located along the dashed line in (c)). e) schematic drawing of an ICO particle observed along the 2‐fold symmetry axis (which has been azimuthally located such a family of *(011)_RHO_* planes becomes aligned with the experimental angle of the dashed line in (c)). Note that this simple model predicts the angular distribution of legs (sitting on ICO apexes) in excellent agreement with leg positions (a). f) 3D scheme showing the deduced correlation between icosahedral core and leg orientation to yield the nanostar image measured for NS‐4. See text for explanations.

Nanostars NS‐5 and NS‐6 images show major leg groups forming an azimuthally organized distribution that suggests an underlying symmetry. In contrast, the NS‐4 legs configuration is irregular as if some major legs have not grown or been formed properly. The distribution of legs is highly anisotropic; however, it seems to present a mirror plane (m) close to the horizontal direction, and the angle between legs #1 and #2, is identical to the one between legs #3 and #4 (Figure 4a). We must note that the mean DP from the core displays a spot arrangement that roughly follows a 2‐fold axis distribution (elongated hexagon spot configuration, Figure 4b,c). The VDFs extracted from different diffraction spots (A‐D) indicate a polycrystalline star core (Figure 4d), however no VDF shows the typical sand clock pattern expected for an ICO core. Considering that the NS core main symmetry axis may not be perfectly oriented along the electron beam direction, it is reasonable to deduce that leg configuration (mirror plane) and core symmetry (2‐fold axis in DP) may be related.

As both NS‐5 and NS‐6 have been shown to exhibit icosahedral cores, it is natural to test a similar core structure as a first trial input to explain NS‐4 leg configuration and core diffraction pattern, because icosahedral symmetry includes 15×2‐fold rotation axes [43]. The two most intense spots in Figure 4b,c (marked A) correspond to *{011}_RHO_* planes [28], indicating a real space direction where the atomic planes must be closely oriented along the incident electron beam direction. Figure 4e shows a schematic ICO particle oriented along the 2‐fold axis; the particle shows 2 mm planar(2D) point‐group symmetry, displaying the two symmetry operations indicated by ADF image (m) and core diffraction pattern (diad). Also, the ICO apexes follow an azimuthal distribution with angle differences (58° & 64° degrees) very close to experimental measurements of interleg angles (57° and 68°). Choosing the azimuthal angular position of this ICO particle such that a *(011)_RHO_* family of planes is aligned with the experimentally deduced position (dashed line in Figure 4c), the azimuthal leg directions are in full agreement with the ICO apex in the 2D projection (Figure 4a–e). By extending the direction defined by ICO apexes into 3D space, we can deduce that legs marked #1 & #4 should be out of the plane defined by legs #2 & #3 (dashed red circles in Figure 4f). Due to the projection nature of TEM, we cannot conclude from this simple geometric analysis if leg #1 (or leg #4) is oriented upward or downward (see example for legs marked #4 and #4’ in Figure 4f, respectively). Crystal orientation measurements on NS‐4 legs are presented in Figure S3, and a 3D plot of their configuration in Figure 3d. As previously, due to the 180° duality effects on ACOM results, we have chosen the elevation angle sign to prevent the presence of very small inter‐leg angles (around 20–30 degrees). We have included the resulting interleg angular distances in Table 1. The asymmetric profile of NS‐4 and the absence of VDFs with sand clock contrast suggest a highly defective ICO particle at the NS center, which has not allowed the nucleation and growth of decahedral legs on some regions of the NS core

We would like to emphasize that the small protuberance visible on NS‐4 (indicated as leg #5 in Figure 4a) contains pixels with a DP that could be easily indexed by the ACOM software (*[1 ‐3 1]_BCO_* direction, see Figure S3). For a *BCO* crystal observed along this direction, the *[010]_BCO_* vector (leg orientation) shows a rather high elevation angle (∼60°). By measuring the extension of this short leg in the images (Figure 1b) and considering the high elevation angle, we calculate a ∼37 nm leg length. This leg is considerably shorter compared to the other star legs of NS‐4 (#1–#4, which are 60–70 nm long), possibly due to its quite different elevation angle (Table 1). We hypothesize that this leg may have undergone a different nucleation or growth process than the other NS legs (#1–#4).

In brief, precise structural information of complex nanostars was determined by analyzing STEM images, core diffraction patterns, and legs elevation angles. All experimental observations from the three studied NSs can be consistently interpreted by considering ICO cores, where leg positions and spatial orientations correspond to the locations of icosahedral apexes. These corners, formed by the junction of five tetrahedra (or a five‐fold axis), provide ideal sites for decahedral leg epitaxial growth.

A geometrically perfect icosahedron includes five‐fold axes located at apexes and homogeneously distributed in space over the 4π SR solid angle. The angular distances between these axes in 3D space may merely show three different values: a) 64.3 degrees between first nearest neighboring apexes; b) 116.6 degrees between second nearest neighboring apexes; and finally, c) 180 degrees for five‐fold axis that are diametrically opposed.

We must emphasize that measurements of inter‐leg angles in space provide us with a very stringent test to corroborate the consistency of the structural model based on the ICO symmetry. Table 1 shows the comparison of geometrically expected ICO apex distances and measured inter‐leg angular distances for the NSs. The upper right sector displays values deduced from icosahedral geometry, while the lower left sector displays experimental ACOM measurements. A quick look at Table 1, easily reveals that although the NSs display quite different morphologies, there is an excellent agreement with ICO symmetry. This provides robust support to the direct relation between ICO apex distribution in space and NS leg configuration.

At this point, it is essential to note that 4D‐STEM PED diffraction mapping and ACOM contribute significantly to turning the analysis into a quantitative evaluation, because the legs distribution in space is obtained as a vectorial quantity.

To provide greater robustness and reliability to the results discussed above, it is essential to compare the deduced leg spatial distribution with other recognized methods to derive the nanoparticle 3D dimensional morphology: STEM electron tomography.

**FIGURE 5:**
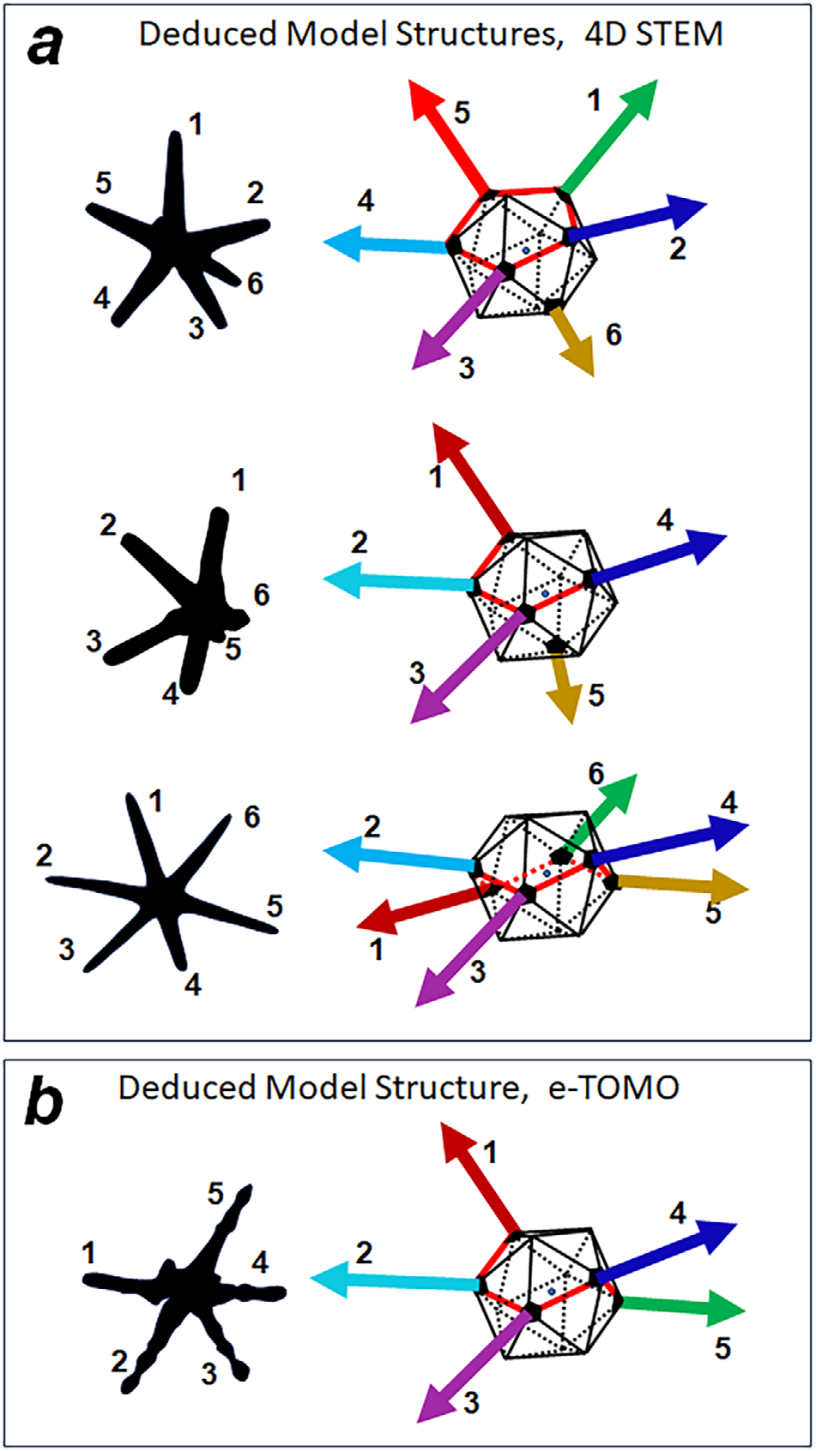
Geometrical schema of legs distribution in space for nanostars formed from icosahedral cores: a) configurations deduced from 4D‐STEM diffraction results (note that results of NS‐6 star (6 leg NS at the bottom of the figure) are based on the measurements of Corrêa et al., [28]. b) Legs configuration for a 5‐leg star (NS‐T) deduced by interpreting tomographic reconstruction reported by Tsoulos et al., [15], see details in Figure S5).

Several publications have already reported STEM ADF tomography of similar NS systems [14, 15]; however, the star 3D reconstructions have not been quantitatively analyzed to identify the geometrical pattern of leg configuration. A very instructive and interesting result has been reported by Tsoulos et al. [15], who presented the tomographic reconstruction of an NS with 5 legs (hereafter noted NS‐T) when observed along the three cartesian axes (see Figure 3 in REF. [15]; the binarized 2D profiles of these images are included in Figure S5b). By following leg positions in each of the three projected images, it is possible to deduce the 3D leg distribution of NS‐T (Figure S5b). The leg configuration is in full agreement with the observed icosahedral symmetry deduced from 4D‐STEM maps. To make the comparison easier, the ideal geometric leg patterns of these 4 solved leg configurations are shown in Figure 5. The use of 4D‐STEM diffraction mapping provides simultaneous information of NS morphology, leg, and core atomic structure in a quantitative and vectorial measurement. Even if crystallographic analysis is, at first sight, less intuitive than atomic resolution images, this greatly simplifies an effective and unambiguous determination of the correlation between those structural features.

Finally, we must emphasize that knowledge of NS structure and shape has been derived from 4D‐STEM diffraction acquired using a total electron dose with a maximum value in the 100–200 e^−^Å^−2^ range. This contrasts with the values required for a STEM ADF ET, where doses are several orders of magnitude higher (10^5^–10^6^ e^−^Å^−2^). 4D‐STEM methods have been confirmed to play a key role in low‐dose structural analysis of beam‐sensitive materials. The most fragile region of NS are the high‐aspect ratio legs, which could collapse or become shorter due to beam bombardment effects. Previous reported results [15] show the occurrence of a pearling of the legs during the acquisition of ADF electron tomography data, a method requiring long electron irradiation of the sample (∼30 min). The pearling of NS legs can be easily recognized in the tomographic reconstruction shown in Figure S5. Even when several diffraction maps have been acquired on the same star, anticorrelation images have always revealed the internal twins along the whole NS legs and no paring of legs has been observed [28], which makes us confident of the reliability of the derived results.

The four different multi‐branched NS show a leg configuration based on the ICO symmetry. Diffraction mapping has yielded unambiguous proof that legs grow from apexes (or location of five‐fold axis) of an ICO core, confirming that the symmetry of the icosahedral core determines the 3D space distribution of the legs. In contrast to the spherically distribution of apexes in icosahedra, major legs seem to grow from apexes following a line located close to a plane (see Figure 5, where a red line following neighboring legs is indicated over the icosahedral cores): a) legs 1–5 in NS‐5; b) legs 1–4 in NS‐4; c) legs 1–6 in NS‐6 and d) legs 1–5 in the NS‐T. Our experimental results also show that when a leg is formed away from the above‐mentioned plane (leg #6 in NS‐5 or leg #5 in NS‐4), it is clearly shorter and thinner than the major legs. This suggests that it has been generated during a secondary growth step, likely triggered by a slightly different phenomenon. These observations provide additional support to the hypothetical growth model suggested by Corrêa et al. [28], who speculated that the growth of legs for icosahedral apexes is initiated by the evolution of a crack in the surfactant layer over the icosahedral core.

**FIGURE 6:**
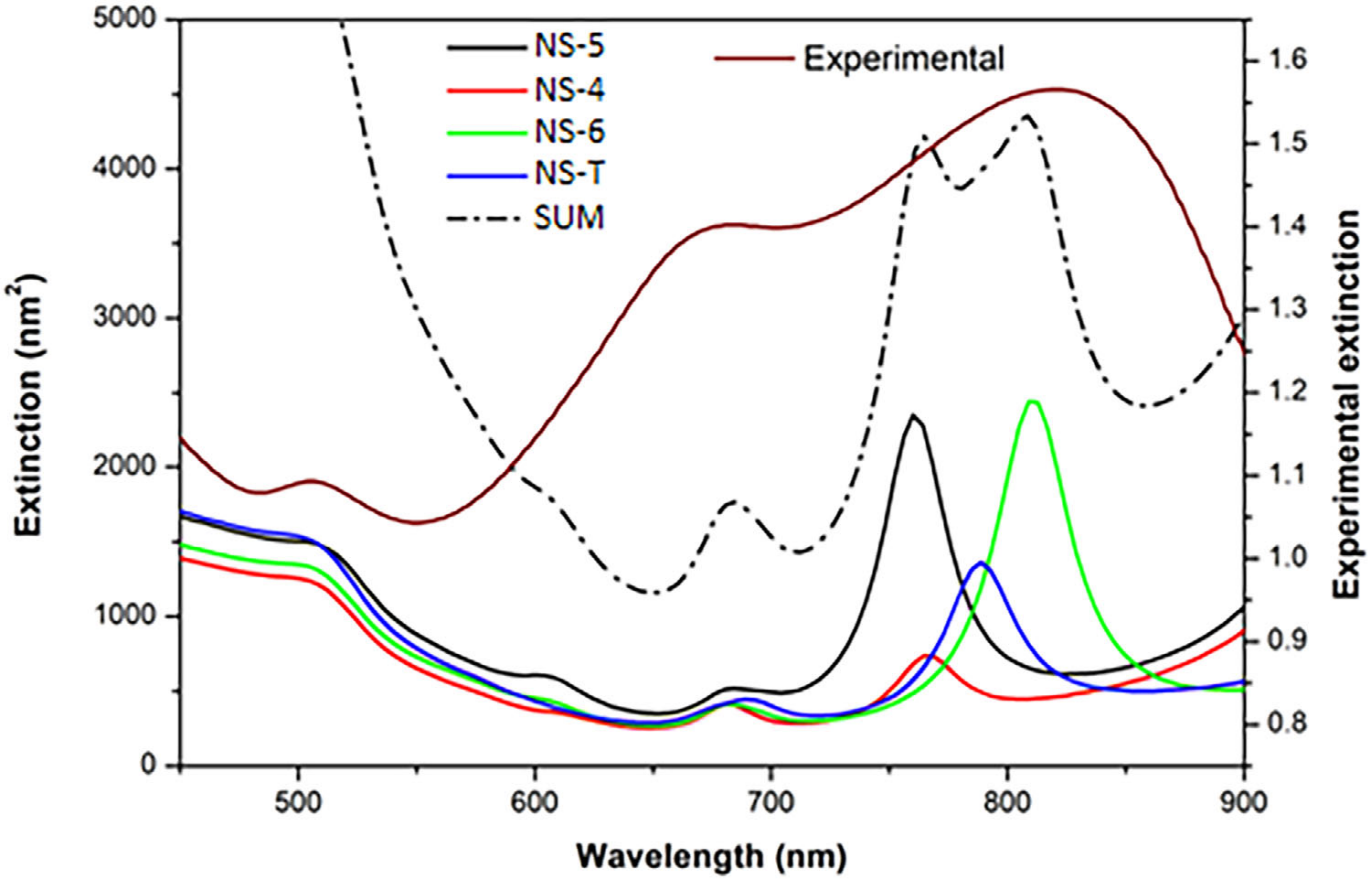
Comparison between the BEM‐simulated extinction spectra of nanostars NS‐5, NS‐4, NS‐6, and NS‐T with the experimental extinction spectrum of the synthesized AuAg nanostar colloid extracted from REF. [50]. The figure also includes the sum of all simulated spectra, which improves the correlation with the experimental data.

As we have already obtained a detailed measurement of the 3D shape of different NSs, it is essential to apply these results to improve our understanding of their optical response and application as plasmonic antennas. Figure 6 shows the Boundary Element Method (BEM) [46, 47] simulation of extinction spectra for the investigated AuAg nanostars. In these simulations, discussed in depth in REF. [54], an approximation was made by considering the NSs to be composed exclusively of Au, neglecting the Ag contribution given its presence in lower amounts, and thus not significantly contributing to the plasmonic response. The larger legs were modelled with lengths of 65 nm (measured from the core center), while the shorter legs, observed in NS‐5 and NS‐4, were assigned to a length of 40 nm.

**FIGURE 7:**
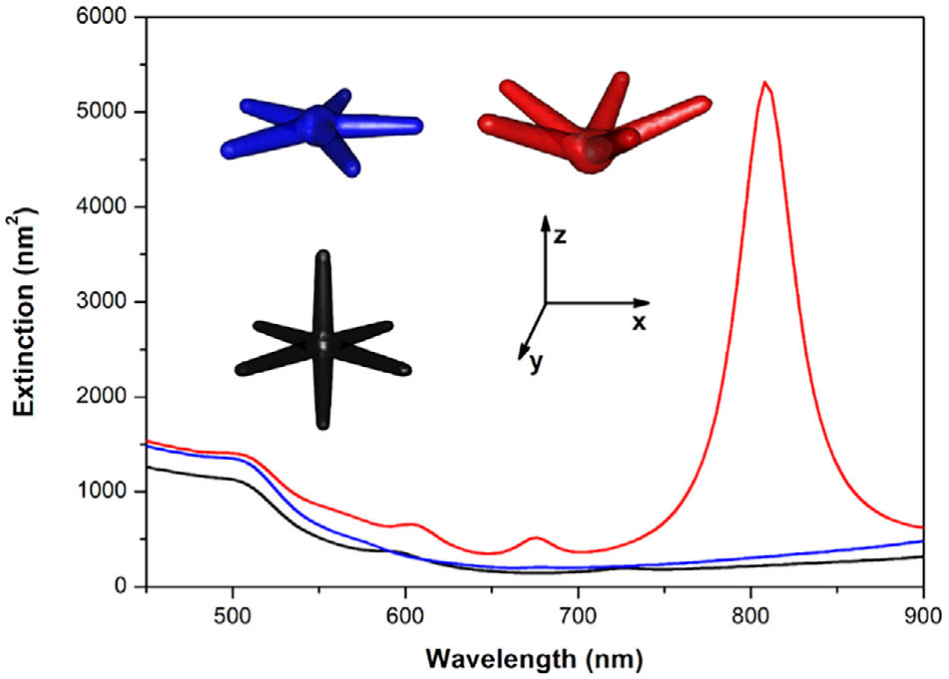
BEM‐simulated extinction spectra for model Au nanostars with different leg orientations: six legs oriented along an octahedral geometry (black), five legs arranged in a planar pentagonal structure (blue), and five legs oriented according to the vertices of an icosahedron (red). Schematics of the leg orientations are shown in the inset for each model.

All nanostars exhibit two main plasmonic resonances in the range of 650–850 nm, showing very good agreement between the experimental and simulated extinction spectra, particularly when comparing the experimental data with the summed contributions of all four simulated structures. The broad experimental bands reflect the structural diversity of the synthesized sample, especially variations in the number, orientation, and length of the legs. Nevertheless, the overall agreement between experiment and simulation supports the interpretation that the observed optical properties are a direct consequence of nanostar morphologies derived from an icosahedral core. To further demonstrate this relationship, additional BEM simulations were carried out for three model nanostars, whose extinction spectra are presented in Figure 7. The models correspond to: (i) a six‐leg Au NS with legs oriented along the vertices of an octahedral structure (black curve); (ii) a five‐leg Au NS with all legs lying in the xy‐plane, i.e., a planar configuration expected for a NS build around a decahedral core (blue curve); and (iii) a five‐leg Au NS with legs oriented according to the vertices of an icosahedron (red curve).

Given the leg dimensions and their high aspect ratios, plasmonic resonances are expected to appear in the near‐infrared region. However, to facilitate direct comparison with the experimental spectra, the discussion here is restricted to the 400–900 nm range. Within this spectral window, the only significant feature observed for the octahedral and planar structures is a peak around 510 nm, most likely corresponding to plasmonic contributions from the core and to transverse modes perpendicular to the leg axes. Very small intensity peaks can also be found in the range 500–800 nm, which were previously assigned to harmonic peaks associated to a propagating plasmon mode [48] (see Supporting information, Figure S6). None of these structures can reproduce the experimental spectral profile, suggesting that the optical response of the synthesized nanostars cannot be properly described by configurations in which all legs lie within the same plane. To illustrate this effect, Figure 7 also includes the extinction spectrum of a five‐leg nanostar whose legs are tilted from the xy‐plane toward orientations consistent with the icosahedral vertex directions. This configuration yields a spectral profile in excellent agreement with the experimental data (peaks at ∼520, 600, 680, and 800 nm), reinforcing the key role of icosahedral symmetry in determining the plasmonic behavior of these nanostars. An in‐depth investigation of the optical properties of such nanostar is discussed in Ref. [54].

## 3 Conclusions

We have shown that the seeded synthesis of branched NS with high aspect‐ratio legs is based on the icosahedral symmetry of the cores. Our study reveals the wide range of structural information available from a single 4D‐STEM diffraction map whose acquisition lasted a few tens of seconds: i) leg azimuthal positions from STEM images; ii) NS core symmetry by electron diffraction patterns; and iii) leg elevation angles (crystal orientation measurements). This highlights the high potential of the 4D‐STEM diffraction map for the planning of in situ experiments, where major structural changes can occur in a short span of time. We have analyzed NS with different numbers of legs and apparently different 3D spatial configurations; all NSs showed legs (decahedral wires) located at the vertex (five‐fold axes) of an ICO central core. Legs are distributed in space with a well‐defined tendency to be anchored on neighboring apexes of the icosahedron, which are located closer to the plane, but not in a fully planar distribution. The non‐planar position of legs defined by the ICO symmetry induces an optical response generating an infrared peak at ∼800 nm that would be absent for planar or octahedral leg distribution. This reveals the high potential of NS for optical enhancement applications in the near infrared region. Our results support the growth model where legs are sequentially formed on neighboring ICO apexes by a crack propagation of the surfactant layer covering the NS seed or core [28]. This provides an essential new understanding that will be extremely useful to optimize and refine the synthesis of noble metal nanostars.

## 4 Experimental Section/Methods

### 4.1 Specimen Preparation

The high aspect‐ratio leg nanostars were synthesized by seed‐mediated synthesis. Firstly, nanometer‐sized Au seed particles were prepared by adding an ice‐cold solution of NaBH4 (0.6 mL, 0.01 m) into an aqueous solution of HAuCl4 (10 mL, 0.25 mm) and Triton X‐100 (0.15 m). The mixture was stirred for 2 min and left for 10 min at 4°C. Subsequently, 0.4 mL of 25 mm HAuCl4 solution was added to a 20 mL Triton‐X solution (0.15 m), followed by ascorbic acid (1.2 mm), AgNO3 (100 µm), and Au seeds (ranging from 0.06 nm) were added to the growth solution and stirred for 10 min. The solution was centrifuged for 10 min at 3500 g, and Ultrapure MilliQ water (18.2 MΩ cm) was used to disperse it to a final concentration of approximately 2 nm. Transmission Electron Microscope (TEM) samples of nanostars were prepared by drop casting the solution onto a holey carbon grid (Figure S1).

### 4.2 Electron Microscopy Experiments

The Scanning TEM data and electron diffraction mapping (4D‐STEM) have been acquired using a probe‐corrected Thermo Fischer Spectra 300 microscope. Electron diffraction patterns were recorded using a direct detection camera (DDC, Quantum Detector Merlin 256 × 256) at 300 KV. Precession electron diffraction (PED) has been performed with a precession angle of 1 degree and a frequency of 1 kHz (Nanomegas hardware and TopSpin software). The 4D‐STEM data has been acquired using a total dose of ∼100–200 e^−^Å^−2^ per frame (2 nm‐wide probe; 1.0 mrad half convergence angle; pixel step 1 nm; dwell time 1 ms).

### 4.3 Derivation of NS Images (VDFs) from 4D‐STEM Maps

Different kind of images can be calculated from a 4D‐STEM diffraction map: a) virtual annular dark field (VADF), where the intensity is calculated by adding all pixels inside an annular region of each diffraction pattern; b) VDF images, built by calculating for each pixel the intensity associated with selected diffraction peaks, such that the image contains the spatial distribution of pixels generating a particular diffraction spot (see Figures 2 and 3); finally c) anticorrelation images, where contrast is generated comparing a diffraction pattern with its nearest neighbors to reveal crystal grain boundaries and twins.

The anticorrelation image value *C(x,y)* associated to a generic pixel *p(x,y)* is derived by calculating: [44]

(1) $$C\;\left(x,y\right)=\sqrt{\frac{\sum_{i,j}{\left[p_{x,y}\left(i,j\right)-\;p_{x+1,y}\left(i,j\right)\right]}^{2}+{\left[p_{x,y}\left(i,j\right)-\;p_{x,y+1}\left(i,j\right)\right]}^{2}}{2n}}\;$$

where *i,j* is the coordinate of pixels in a measured ED, with n total pixels. The contrast is low from homogeneous regions and attains the highest values when the ED patterns change drastically from pixel to pixel (see Figure 1).

### 4.4 Details of 4D‐STEM Diffraction Data Processing and Simulation

Automatic crystal Orientation mapping (ACOM) analysis was based on the Pyxem open software included in the Hyperspy open package [49]. This software uses a conventional template matching by comparing the experimental pattern with a library of ED simulations. Given the quality of PED patterns, we estimate that the angular resolution of the pattern matching results is limited to ∼2 degrees, as typically obtained in PED‐based ACOM. We would like to comment that the application of ACOM to the NS legs represents a challenging case, because we are dealing with thin crystal domains (thickness 5–7 nm), that generate large excitation errors, with the consequence that diffraction beams can be excited with a large orientation mismatch (<5°) even from ideal diffraction conditions. All diffraction patterns used for each leg orientation analysis are shown in Figures S2 and S3, where we may note that most of them are PED patterns of high‐quality showing spots forming arrays of high symmetry.

### 4.5 Nanostar Core and Leg Atomic Structure

Nanoparticles from noble metal nanoparticles can form multi‐domain structures with the peculiarity of containing a five‐fold symmetry axis; these structures are usually described as multiply‐twinned particles (MTPs) [44]. This description involved two different structures: a) Decahedron (DEC), formed by the junction of 5 tetrahedra along the five‐fold axis; and b) Icosahedron (ICO), formed by 20 tetrahedra connected at one tip. Although noble metals display face‐centered cubic stable phases, the need to fill space (without gaps between tetrahedra) leads to a structural deformation of the cubic lattice [44], which results in a Body Centred Orthorhombic (*BCO*) lattice for DEC particles and a Rhombohedral unit cell (*RHO*) for icosahedral ones [28, 45]. The ED pattern library used for determining the NS leg orientation in space has been built considering a *BCO* unit cell (a = 0.265 nm, b = 0.271 nm, c = 0.358 nm, space group = Immm, #71).

### 4.6 Boundary Element Method (BEM) Simulations

The optical response of Au nanostars was simulated using the Boundary Element Method (BEM) [46], as implemented in the MNPBEM17 toolbox [47]. As an approximation, all nanoparticles were modeled as composed exclusively of Au, neglecting the presence of Ag. The dielectric function of Au was taken from experimental data compiled by Johnson and Christy [51], while the surrounding medium was modeled as water, with a constant refractive index of 1.333. The nanostar core was modeled with a sphere with a diameter of 25 nm, while the legs were described by cones with base and tip diameters of 15 and 10 nm, respectively. To represent the experimentally observed morphologies, two leg lengths of 65 and 40 nm were used to describe the longer and shorter branches, respectively. The surface meshes, consisting of approximately 7000 triangular elements, were generated using R [52] and Meshlab [53] software. To reduce computational cost associated with solving large systems of boundary equations, the hierarchical matrix solver [54] implemented in MNPBEM17 was employed. Simulations were conducted for an incident electric field polarized along the *x, y*, and *z* directions. The reported spectra correspond to the average over these three polarization configurations.

## Funding

EPSRC (EP/R008779/1). Busch Biomedical Grant Program at Rutgers University. FAPESP (2024/00998‐6, 2025/05239‐9, 2016/21070‐5, 2022/11983‐4). CNPq (402676/2021‐1, 303025/2022‐0, 403359/2023‐6, 402092/2025‐2, 140596/2020‐8, 405087/2021‐7). CAPES (1765876/2018). ERC, European Union's Horizon 2020, 865819.

## Supporting information




**Supporting File**: smll73216‐sup‐0001‐SuppMat.pdf.

## Data Availability

The raw data utilized in the work will be accessible from a repository in our university, providing a DOI. The manuscript is accompanied by a supplementary information file with details about methods and data analysis.
