# Supplementary material for: Essential Role of Icosahedral Symmetry in the 3D Shape of AuAg Plasmonic Nanostars With High Aspect‐Ratio Legs
Source: Small. 2026 Mar 29;22(27):e73216. doi: 10.1002/smll.73216 (PMC13173317; doi:10.1002/smll.73216)
Supplement: Supplementary file 1 — Supporting File: smll73216‐sup‐0001‐SuppMat.pdf. [file SMLL-22-e73216-s001.pdf]

Essential Role of Icosahedral Symmetry in the 3D Shape of AuAg Plasmonic Nanostars with High Aspect-ratio Legs

Leonardo M. Corrêa,[1] Simon M. Fairclough,[2] , Kaleigh M. R. Scher,[3] , Supriya Atta,[4] , Diego P. dos Santos,[5], Caterina Ducati,[2], Laura Fabris,[6], Daniel Ugarte[1*]

1 Instituto de Física Gleb Wataghin, Universidade Estadual de Campinas; Campinas, 13083- 859, Brazil.
2 Department of Materials Science and Metallurgy, University of Cambridge; Cambridge, UK.
3 Department of Materials Science and Engineering, Rutgers University; Piscataway, 08854, USA.
4 Department of Biomedical Engineering, Duke University; Durham, NC 27708, USA.
5 Instituto de Química, Universidade Estadual de Campinas, Campinas, 13083- 859, Brazil.
6 Department of Applied Science and Technology, Politecnico di Torino; Turin, 10129, Italy.

* Corresponding author. Email: dmugarte@ifi.unicamp.br, dugarte@unicamp.br

Content:

Gathering nanostar structure and morphology from 4D-STEM diffraction maps

Table S1. List of leg orientation in space for NS-1 and NS-2 deduced by Automatic Crystal Orientation Mapping (ACOM).

Figure S1: Low magnification TEM images of NSs.

Figure S2: Diffraction patterns used to determine leg orientation for NS-1.

Figure S3: Diffraction patterns used to determine leg orientation for NS-2.

Figure S4: Schematic geometric draw of leg spatial distribution for NS-1.

Figure S5: Leg configuration on particles NS-3 and NS-T

Figure S6: BEM-simulated extinction spectra for model Au nanostars with six legs (octahedral geometry).

References

Gathering nanostar structure and morpholohy from 4D-STEM diffraction maps

The information on NS structural properties is derived from: i) leg positions in STEM images (2D, azimuthal data); ii) core diffraction patterns; and iii) leg elevation angles (3D configuration).

NS-5 exhibits several indications that a 5-fold symmetry axis is significant for understanding its atomic arrangement. Firstly, TEM and STEM images reveal five primary legs, each separated by about 72 °, along with an additional, shorter leg positioned at an azimuthal angle halfway between the main legs (~36° from each major leg). NS-5 is one of the dominant morphologies in our samples and has also been a frequent leg configuration in previous studies [1,2]. The high-intensity lines in the ACM (anti-correlation) image indicate twin defects along the legs that join at core center (Figures 1a & 2a). This highlights a well-defined relation between the spatial orientation of the legs and the structure of the star core.

Secondly, the core diffraction pattern exhibits a distinct 5-fold rotation axis parallel to the incident beam direction (z-axis), which represents an unusual rotational symmetry in crystallography. In nanoparticle research, two categories of multiply twinned particles (MTPs) —decahedra (DEC) and icosahedra (ICO)— show this symmetry. Differentiation between these forms requires analysis of virtual dark fields (VDFs) from diffraction spots, specifically focusing on the presence of sand clock contrast. The combination of a five-fold rotational axis and sand clock VDF is indicative solely of an icosahedral core. For NS-5, only a diffraction spot (marked H in Figure 2) generates a VDF image with a clear sand clock pattern. Since not all VDFs display this contrast, it is essential to evaluate supplementary experimental evidence.

At this stage, it is necessary to examine the occurrence and position of the 6th leg observed in TEM or STEM images. When considering a decahedral core, previous studies have proposed that legs emerge from twin planes aligned parallel to the 5-fold axis [1,2]. Nevertheless, a DEC model does not easily account for, or predict, the specific attachment site of a sixth leg. In contrast, an icosahedral core model offers a more robust explanation for both the presence and azimuthal position of the 6th leg. As illustrated in Figure S4, ICO particles consist of two stacked decahedra (each formed by the assembly of five tetrahedra), rotated by 36° ($2\pi/10$ rad) about the five-fold axis. If leg attachment points are situated on the ICO apexes, it becomes possible to position five primary legs on the apexes of one decahedron, while the sixth leg attaches to an apex from the second, rotated decahedron. This configuration naturally results in the sixth leg appearing at a 36-degree offset from the primary legs when the NS is viewed along the 5-fold axis (see Figure. S4).

To finish analysing the NS-5 experimental data, we need to examine the leg elevation angles determined from the precession electron diffraction (PED) patterns at individual pixels (Figure S2 and S3). Experimental data shows that all six legs of NS-5 have similar elevation angles, ranging between 17° and 22° (see Table S1). According to the DEC-based NS model, 5x legs are predicted to emerge from twin planes, growing perpendicular to the 5-fold axis; that is, with zero elevation angle relative to the sample plane [2]. None of these legs are perfectly perpendicular to the 5-fold axis as would be expected for a decahedral core. When considering the two stacked decahedra inside the ICO particle illustrated in Figure S4, we should observe that five legs have an elevation angle of 26.6 degrees in one direction, while the sixth, shorter leg in NS-5 should display an identical magnitude but with opposite sign.

It is important to note that, because electron diffraction is two-dimensional in nature (projection geometry), PED diffraction analysis provides the absolute values of leg elevation angles but cannot determine their sign (i.e., whether legs are pointing upwards (+z) or downwards (−z)). This inherent limitation is known as the 180° duality in Automatic Crystal Orientation Mapping (ACOM) [3]. In line with the ICO core model discussed above (Figure S4), the five primary legs should have elevation angles in the same direction (downwards, protruding from the lower ICO apexes), whereas the sixth leg points in the opposite direction (upwards, apex located on the top decahedron of the ICO particle). When the elevation angles are set according to this structural model, all interleg spatial angles show an excellent agreement with the values of an icosahedral symmetry (see Table 1).

Briefly, an ICO core can readily explain the experimental distribution of leg azimuthal and measured elevation angles (see Figure. S4). All three analysed criteria (images, diffraction pattern, and elevation angles) support the presence of an icosahedral core, with legs orientation defined by 5-fold axis located at ICO apexes. These criteria are difficult to fulfil with a decahedral core (the alternative particle structure with a 5-fold axis).

To continue the analysis of images and diffraction patterns of NSs, we will discuss below the ensemble of experimental results acquired for the rather asymmetrical NS-4 (displaying 4 major legs). As previously, we will make a sequential analysis of leg azimuthal positions in STEM images, core diffraction patterns and finally, leg elevation angles.

Firstly, VADF images (Figure 1b and 4a) reveal that the NS-4 legs have a mirror plane (m) perpendicular to the line between legs #1 and #4. The angles between legs 1-2 and 3-4 are both about 57°, while angle 3-4 is slightly larger at ~68°. Secondly, the core diffraction pattern suggests a 2-fold rotation axis (Figures 4b and 4c). VDF images only confirm that the core is polycrystalline and lack the sand clock pattern expected for a perfect icosahedral core (Figure 4d).

Since NS-6 and NS-5 structures have been attributed to an icosahedral core, and ICO particles also display 2-fold rotational axis, we will examine if an ICO particle along this axis could be used to interpret the available experimental data. Just looking at an ICO particle perfectly aligned along 2-fold axis, we may note that, the schematic draw (Figure 4e) indicates a 2mm planar (2D) point-group symmetry, displaying the two symmetry operations indicated by ADF image and core diffraction pattern. Also, the ICO apexes follow an azimuthal distribution with angles differences (58° & 64° degrees) very close to experimental measurements of interleg angles (57° and 68°). The stronger diffraction spot (marked A in Figure 2c) corresponds to the $(011)_{RHO}$ planes (*RHO*, Rhombohedral); using the azimuthal orientation of these planes (dashed line in Figures 3c and 3e), we may determine the azimuthal position of the ICO core. This simple geometrical construction shows an ICO apex arrangement to match the experimental NS-4 leg positions (Figure 4a), what justifies a deeper analysis of an ICO core to explain NS-4 structure.

The next step includes the comparison of leg elevation angles with the icosahedral apex vertical positions (3D leg configuration). Analysing apexes positions with the ICO core observed along a 2-fold axis, legs #2 and #3 should lay in the *xy* plane (zero elevation), while legs #1 and #4 should be out of the *xy* plane at ~32° elevation (either up or down; see Figure 3f). As mentioned previously, the leg elevation angles values can be derived from the experimental PED diffraction but not their sign (upwards or downwards are acceptable solutions). We have chosen the sign of elevation angles to avoid the occurrence of very small inter-leg angles (in the 20-30 degrees range) that are never observed in TEM images of NS. This may seem arbitrary, but by applying this simple criterion (see Table S1), we may obtain a leg distribution in space that matches the ICO apex distribution in space (see Table 1) for major legs at the lefts side of the NS (Figure1c and 4), adding further support to an ICO core symmetry for NS-4. The asymmetric profile of NS-4 and the lack of occurrence of sand clock contrast pattern in VDFs suggest the NS core with a highly defective ICO structure, what hinders the growth of decahedral legs at the right side of the NS (Figure 1c).

In summary, the use of an ICO core along a 2-fold axis for NS-4 provides a strong and consistent correlation between the three-dimensional positions of icosahedral apexes and the observed arrangement of legs (see Figure 5).

All three evaluation methods —images, diffraction patterns, and measurements of leg elevation angles— indicate that an icosahedral core is present across the three NS morphologies analyzed. However, as mentioned in the manuscript, not all readers may find interpreting 4D-STEM diffraction mapping straightforward. Therefore, it's important to compare these results with the traditional method for determining nanoparticle morphology: STEM electron tomography. A detailed analysis of tomographic projections along the x, y, and z axes, as reported by Tsoulos et al. [2], is provided in Figure S5. Interestingly, the leg distribution determined

through tomography perfectly matches the NS-4 leg configuration (legs #1 to #4) derived using basic geometric and symmetry principles for an ICO core along the 2-fold axis, as shown in Figure 4f. Specifically, legs #2 and #3 lie in the x-y plane, while legs #1 and #4 show similar elevation angles.

Table S1. Leg length and orientation in space derived from diffraction mapping and template-matching-based automatic crystal orientation [3] to a Body Centred Orthorhombic phase (BCO) phase (PYXEM open software [4]). The in-plane (*xy*) rotation of the legs (starting from x axis) is indicated by the azimuthal angle. The angular distance to the *xy*-plane is measured by the elevation angle (note that in the selected coordinate system *z*-axis points up in the sample plane, opposite the electron beam direction (PYXEM axis configuration [4])

Leg #	**Azimuthal [deg]**	**Elevation [deg]**	**Length [nm]**
	**NS-5 (5-fold symm.)**		
**1**	85.0	22.4	76
**2**	7.2	16.7	61
**3**	-57.8	16.7	59
**4**	-139.0	20.5	64
**5**	153.5	16.7	63
**6**	-32.4	-16.7	46
	**NS-4 (anisotropic)**		
**1**	72.0	0.0	63
**2**	135.7	-14.9	59
**3**	-163.8	16.7	66
**4**	-89.4	45.0	72
**5**	-54.8	-60.9	37

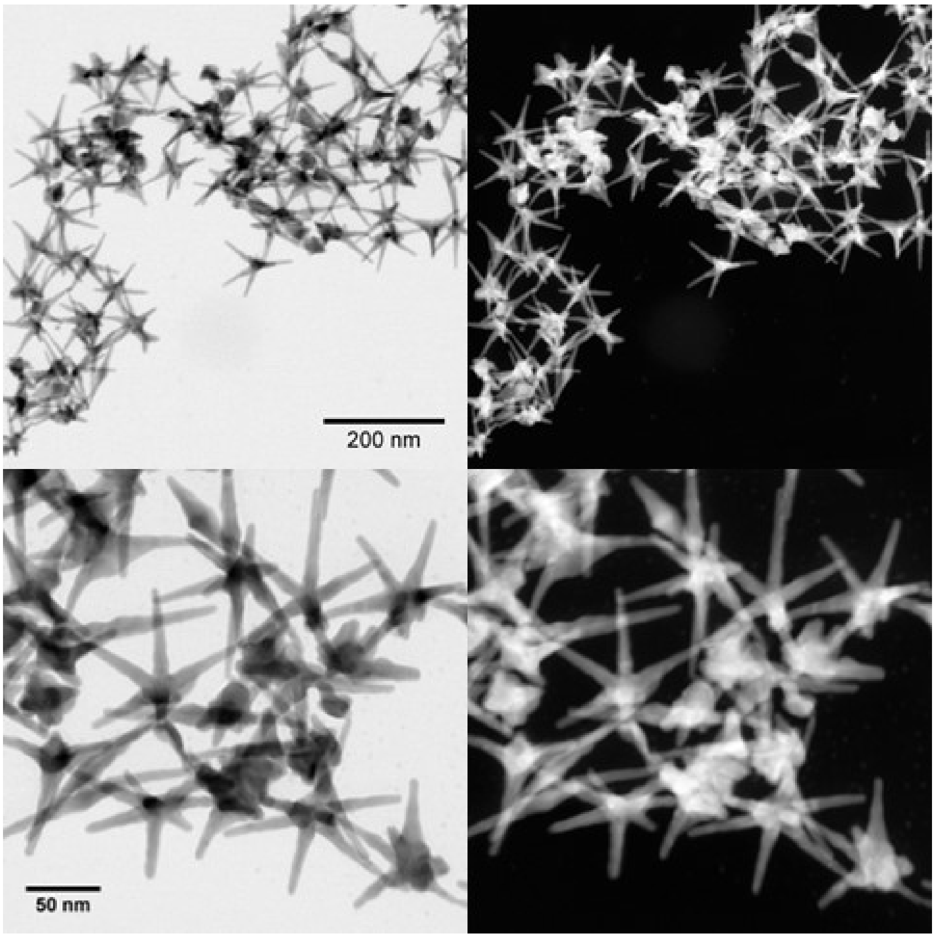

Figure S1. STEM images (Bright Field -BF at left, and Annular Dark Field-ADF at right) displaying the general view of AuAg nanostars synthesized by the seeded method. Note that star legs are 50-70 nm long and their aspect-ratio in the 5-8 range.

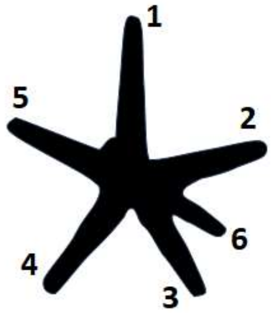

Leg #	Crystal Orientation *[uvw]*
1	[2 $\bar{1}$ 1]
2	[3 $\bar{1}$ 1]
3	[3 $\bar{1}$ 1]
4	[0 $\bar{1}$ 2]
5	[3 $\bar{1}$ 1]
6	[3 $\bar{1}$ 1]

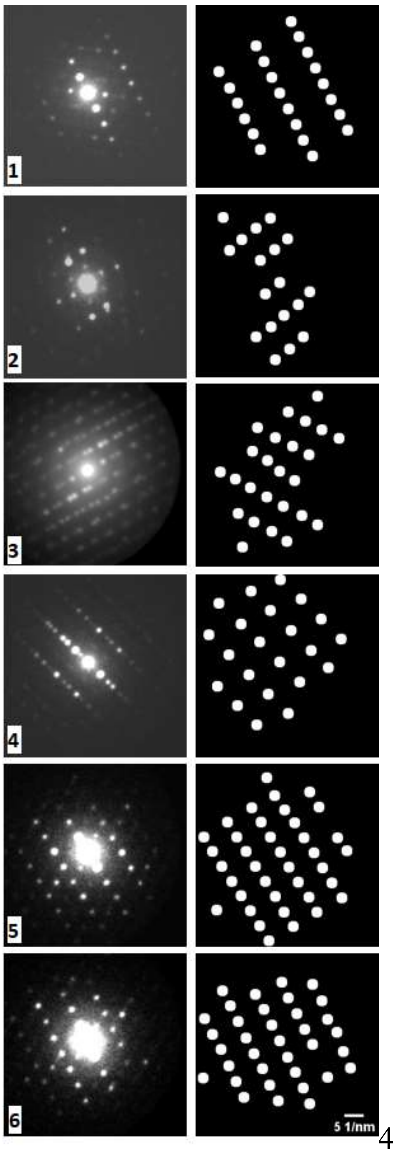

Figure S2. ACOM processing of the 4D-STEM PED diffraction map data to obtain the NS-5 legs orientation in space by applying template matching. The ED patterns used to determine leg orientation are displayed, as well as the geometrical distributions of diffraction spot identified in each diffraction pattern used to create a mask before template matching application. Most of these patterns correspond to single pixel measurement except for Leg #3 where the orientation was derived from the mean leg ED. The Table includes the resulting crystal orientation direction along the electron beam direction as derived using PYXEM software [4]; leg orientation is calculated from the $[010]_{BCO}$ direction (Body Centred Orthorhombic) in space after proper azimuthal rotation.

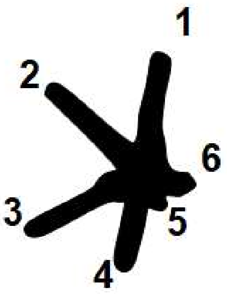

Leg #	Crystal Orientation *[uvw]*
1	$[\bar{2}\,01]$
2	$[7\,\bar{2}\,4]$
3	$[3\,\bar{1}\,1]$
4	$[1\,\bar{1}\,0]$
5	$[1\,\bar{3}\,1]$

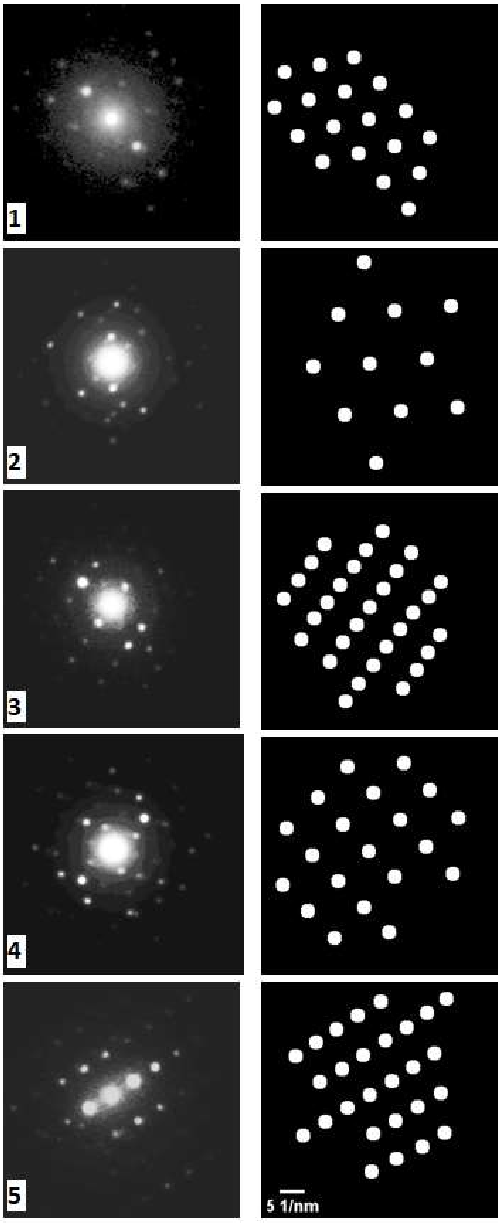

Figure S3. ACOM processing of the 4D-STEM PED diffraction map data to obtain the NS-4 legs orientation in space by applying template matching. The ED patterns used to determine leg orientation are displayed, as well as the geometrical distributions of diffraction spot identified in each diffraction pattern used to create a mask before template matching application; all these patterns correspond to single pixel measurements. The table includes the resulting crystal orientation direction along the electron beam direction as derived using the PYXEM software [4]; leg orientation is calculated from the $[010]_{BCO}$ direction (Body Centred Orthorhombic) in space after proper azimuthal rotation. It is important to emphasize that diffraction patterns from pixels on the small protuberance numbered 5 were easily indexed by ACOM and indicated a 37 nm long leg at an elevation angle of ~60 degrees (this evidence explains why this leg seems so short in the image, essentially a 2D projection).

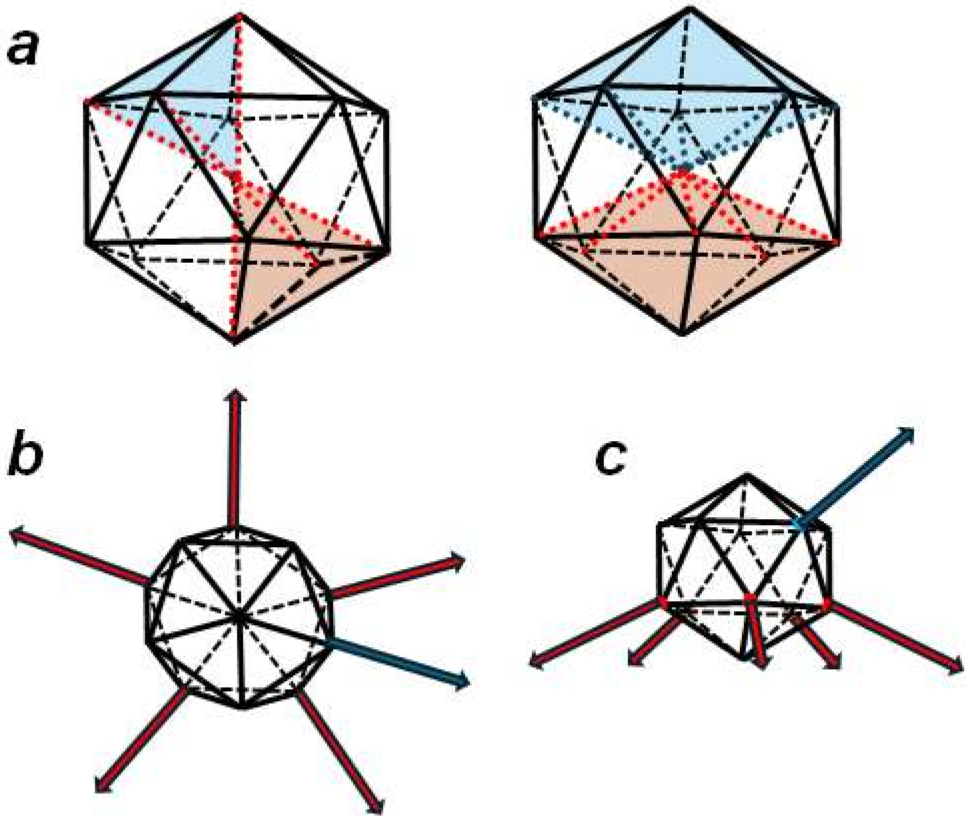

Figure S4. Schematic drawing of the structural aspects associated to organization of the 20 tetrahedral forming an ICO particle, all tetrahedra share a tip at the particle center. a) Each tetrahedral crystallite is associated to a diametrically opposed tetrahedron rotated by 60, but crystal structure of both tetrahedra are identical and perfectly aligned. This originates the expected sand clock contrast in VDFs. Also, tetrahedral crystallites can be grouped to form vertical stacking along the 5-fold axis of two decahedral particles rotated by $(2\pi/10)$. b) when an icosahedron is observed along the 5-fold direct, the directions determined between the particle centre and the five lateral apexes of a decahedron may generate a legs distribution (red arrows at bottom decahedron apexes) with 5-fold symmetry. c) when the 6th leg (blue arrow) is added to the previous structure at an azimuthal angle between the 5-fold symmetry legs (red arrows) this 6th leg must be located at the apex of a second stacked decahedron (upper one) of the icosahedral NS core (see schematic drawing).

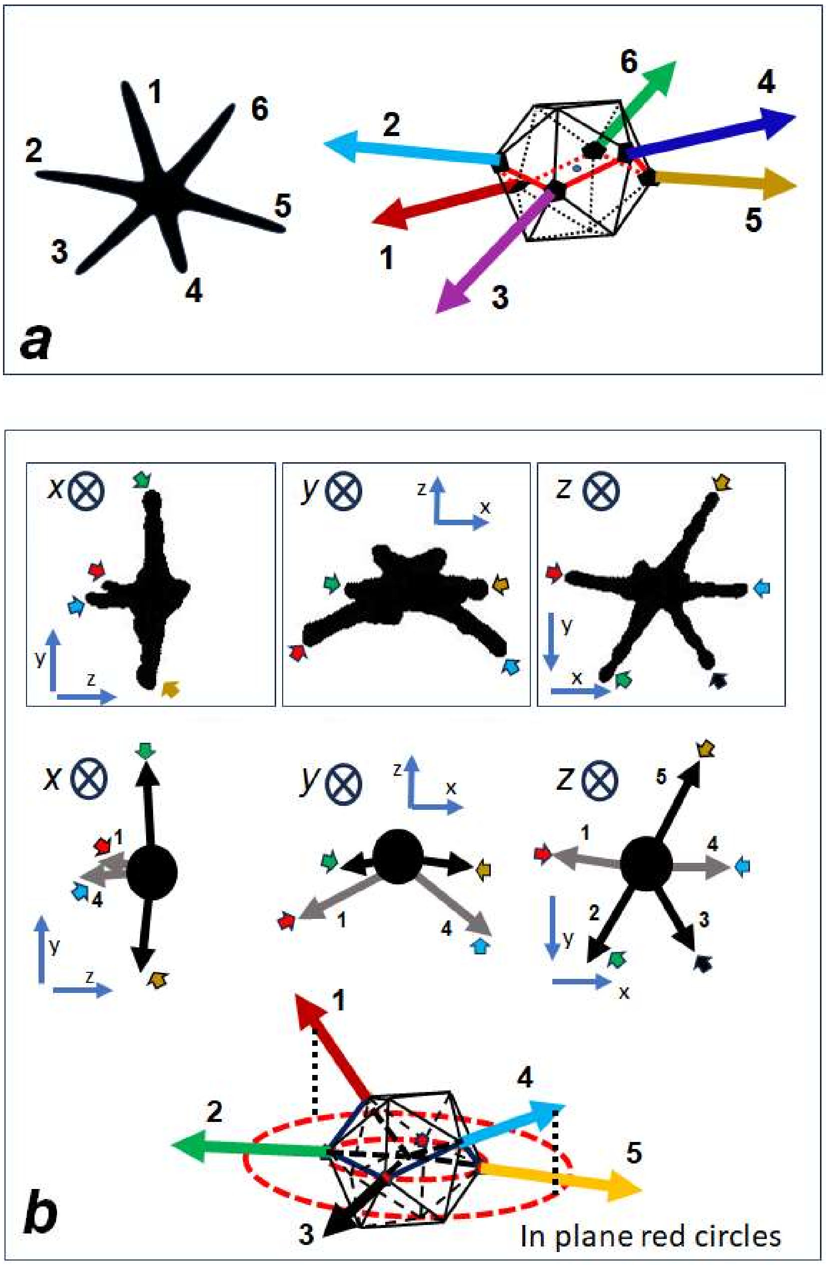

Figure S5. Ideal leg distribution in space when growing from icosahedral cores. These configurations have been deduced from results that have been previously reported based on electron diffraction (a, Corrêa et al. [5]) and using electron tomography data (b, from REF Tsoulos *et al.* [2]). The top of (b) shows the binarized profile of 3D tomographic NS structure reconstruction when observed along different projection axes (*x*, *y*, *z*). The central part displays an easy-to-understand schematic drawing deduced from the projections representing the relation between legs (represented by arrows) and a NS core (represented by the grey sphere). The *z*-projection clearly reveals that the NS is formed by 5 legs, however this fact is difficult to gather when looking at *x*- or *y*-projections. From another point of view, we must emphasize that both *x*- and *y*-projections demonstrate that two legs (indicated by red and blue arrows, numbered #1 and #4 respectively) are out of the x-*y* plane, pointing toward negative *z*-axis. This tomographic 3D reconstruction can be easily explained taking an icosahedral core and a leg distribution in full agreement with results derived from electron diffraction for NS #1-3. Legs indicated 1, 2 and 5 lay on a plane, while legs 1 and 4 show an elevation angle of (31.7 degrees) in relation to that plane (see geometrical scheme at the bottom).

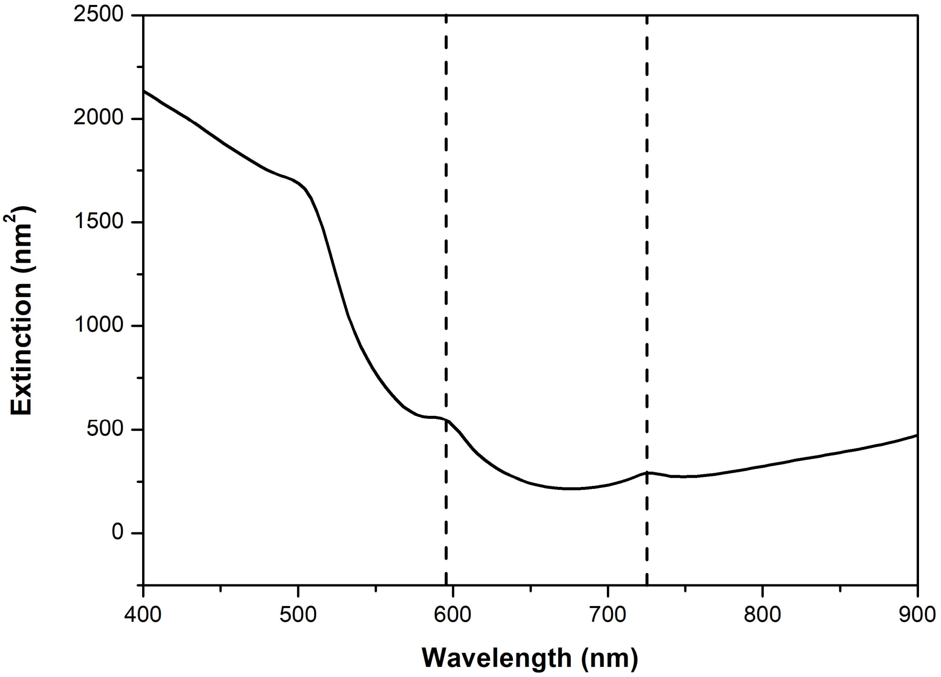

Figure S6. BEM-simulated extinction spectra for model Au nanostars with six legs oriented following an octahedral geometry with incident field polarization along the x-axis. The dashed lines indicate the positions of two very small intensity modes associated with harmonics of a propagating plasmonic mode [6].

References

1. Atta, S.; Beetz, M.; Fabris, L., Understanding the role of AgNO3 concentration and seed morphology in the achievement of tunable shape control in gold nanostars, Nanoscale **2019**, 11 (6), 2946–2958.

2. Tsoulos, T. V.; Atta, S.; Lagos, M. J.; Beetz, M.; Batson, P. E.; Tsilomelekis, G.; Fabris, L. Colloidal Plasmonic Nanostar Antennas with Wide Range Resonance Tunability. Nanoscale **2019**, 11 (40), 18662−18671.

3. Rauch, E. F.; Portillo, J.; Nicolopoulos, S.; Bultreys, D.; Rouvimov, S.; Moeck, P. Automated Nanocrystal Orientation and Phase Mapping in the Transmission Electron Microscope on the Basis of Precession Electron Diffraction. *Z. Kristallogr.* **2010**, 225 (2–3), 103–109.

4. de la Peña, F.; Prestat, E.; Tonaas Fauske, V.; Burdet, P.; Lähnemann, J.; Jokubauskas, P.; Furnival, T.; Carter, F.; Nord, M.; Ostasevicius, T.; MacArthur, K. E.; Johnstone, D. N.; Sarahan, M.; Taillon, J.; Thomas, A.; pquinn, dls.; Migunov, V.; Eljarrat, A.; Caron, J.; Nemoto, T.; Poon, T.; Stefano, M.; actions, u.; Tappy, N.; Cautaerts, N.; Somnath, S.; Slater, T.; Walls, M.; pietsjoh; Ramsden, H. Hyperspy/Hyperspy: V2.0.1; *Zenodo*, **2024**.https://doi.org/10.5281/ZENODO.10709941.

5. Corrêa, L. M.; Fairclough, S. M.; Scher, K. M. R.; Atta, S.; dos Santos, D.P.; Ducati, C.; Fabris, L.; Ugarte, D. Atomic structure and 3D shape of a multibranched plasmonic nanostar from a single spatially resolved electron diffraction map. *ACS Nano* **2024**, 18 (39), 26655–26665.

6. Tsoulos, T.V.; Fabris, L. Interface and Bulk Standing Waves Drive the Coupling of Plasmonic Nanostar Antennas J. Phys. Chem. C **2018**, 122, 50, 28949–28957
